# The O-Ag Antibody Response to Francisella Is Distinct in Rodents and Higher Animals and Can Serve as a Correlate of Protection

**DOI:** 10.3390/pathogens10121646

**Published:** 2021-12-20

**Authors:** Lauren E. Shoudy, Prachi Namjoshi, Gabriela Giordano, Sudeep Kumar, Jennifer D. Bowling, Carl Gelhaus, Eileen M. Barry, Allan J. Hazlett, Brian A. Hazlett, Kristine L. Cooper, Phillip R. Pittman, Douglas S. Reed, Karsten R. O. Hazlett

**Affiliations:** 1 Department of Regenerative and Cancer Cell Biology, Albany Medical College, Albany, NY 12208, USA; cowenl@amc.edu (L.E.S.); gabriela_giordano@hms.harvard.edu (G.G.); 2 Department of Immunology and Microbial Disease, Albany Medical College, Albany, NY 12208, USA; pnamjosh@utk.edu (P.N.); KumarS@amc.edu (S.K.); 3 Center for Vaccine Research, University of Pittsburgh, Pittsburgh, PA 15261, USA; jeb263@pitt.edu (J.D.B.); dsreed@pitt.edu (D.S.R.); 4 MRI Global, Kansas, MO 64110, USA; cgelhaus@MRIGLOBAL.ORG; 5 Center for Vaccine Development and Global Health, University of Maryland School of Medicine, Baltimore, MD 21201, USA; embarry@som.umaryland.edu; 6 Department of Philosophy, Washington University, St Louis, MO 63130, USA; ahazlett@wustl.edu; 7 Department of Ecology and Evolutionary Biology, University of Michigan, Ann Arbor, MI 48109, USA; calcinushazletti@gmail.com; 8 Hillman Cancer Center, Biostatistics Facility, University of Pittsburgh, Pittsburgh, PA 15261, USA; KLC183@pitt.edu; 9 U.S. Army Medical Research Institute of Infectious Diseases, Fort Detrick, Fredrick, MD 21702, USA; phillip.r.pittman.civ@mail.mil

**Keywords:** Francisella, O-Antigen, animal models, rabbits, rodents, humans

## Abstract

Identifying correlates of protection (COPs) for vaccines against lethal human (Hu) pathogens, such as *Francisella tularensis* (*Ft*), is problematic, as clinical trials are currently untenable and the relevance of various animal models can be controversial. Previously, Hu trials with the live vaccine strain (LVS) demonstrated ~80% vaccine efficacy against low dose (~50 CFU) challenge; however, protection deteriorated with higher challenge doses (~2000 CFU of SchuS4) and no COPs were established. Here, we describe our efforts to develop clinically relevant, humoral COPs applicable to high-dose, aerosol challenge with S4. First, our serosurvey of LVS-vaccinated Hu and animals revealed that rabbits (Rbs), but not rodents, recapitulate the Hu O-Ag dependent Ab response to *Ft*. Next, we assayed Rbs immunized with distinct S4-based vaccine candidates (S4Δ*clpB*, S4Δ*guaBA*, and S4Δ*aroD*) and found that, across multiple vaccines, the %O-Ag dep Ab trended with vaccine efficacy. Among S4Δ*guaBA*-vaccinated Rbs, the %O-Ag dep Ab in pre-challenge plasma was significantly higher in survivors than in non-survivors; a cut-off of >70% O-Ag dep Ab predicted survival with high sensitivity and specificity. Finally, we found this COP in 80% of LVS-vaccinated Hu plasma samples as expected for a vaccine with 80% Hu efficacy. Collectively, the %O-Ag dep Ab response is a *bona fide* COP for S4Δ*guaBA*-vaccinated Rb and holds significant promise for guiding vaccine trials with higher animals.

## 1. Introduction

Tularemia (also known as rabbit fever) is a severe disease caused by *Francisella tularensis* (*Ft*), a Gram-negative, facultative intracellular bacterium [1]. While *Ft* can be transmitted by arthropods or direct contact, the most serious form of disease results from inhalation of Type A strains of the bacteria. The infectious aerosolized dose for humans (Hu) is ~15 *Ft* and untreated pneumonic tularemia has a 40–60% fatality rate [2]. It is unclear how *Ft* causes such severe mortality, although it infects many cells, including macrophages, dendritic cells, epithelial cells, and hepatocytes [3]. Lung and liver damage resulting from *Ft* replication and immunopathology is often fatal.

There are currently no tularemia vaccines licensed for Hu use. While disease incidence in the USA is currently low, *Ft* is a Tier 1 select agent due to its historical use as a bioweapon and its ease of aerosol infection; as such, it is a priority for vaccine development. The live-vaccine strain (LVS) was developed in the 1950s from *Ft* subsp*. holarctica* and used as an investigational new drug by USAMRIID until 2019, although reversion to virulence remains a concern [4]. Studies in the 1960s determined that scarification with LVS offers ~80% protection to Hu against low-dose (~50 CFU) aerosol exposure to Type A *Ft* Schu S4 (S4); protection deteriorated with high challenge doses (≥2000 CFU) triggering antibiotic rescue of unprotected Hu [5]. Aerosol delivery of LVS was subsequently shown to be superior to the dermal route; however, Hu protection remained ~80% [6]. No correlates of protection (COPs) were identified in these human studies.

In more recent times, we and others have postulated that live S4-based vaccines would provide better protection against type A strains. We engineered S4 vaccine candidates with unmarked deletions in genes (*guaBA* and *aroD*) encoding critical enzymes in biosynthetic pathways; similar mutations in the enteric pathogens *Shigella* and *Salmonella* Typhi resulted in promising vaccine strains that are safe, highly attenuated, and immunogenic for Hu in clinical trials [7,8,9]. Independently, the Conlan and Sjostedt groups developed and characterized a S4 vaccine candidate, S4Δ*clpB,* which lacks a chaperone protein required for secretion of virulence factors (see [10] and references therein). All three S4-based vaccine candidates (S4Δ*clpB*, S4Δ*guaBA*, and S4Δ*aroD*) have been tested for attenuation and vaccination efficiency (VE) in one or more rodent models (mice (Mo), guinea pigs, rats (Rt)) [10,11,12]. Vaccination of rodents with S4Δ*aroD* or S4Δ*clpB* each provided protection superior to LVS against S4 challenge. Interestingly, S4Δ*guaBA* vaccination in the Mo model did not provide protection, in stark contrast to results in the rabbit (Rb) model. In outbred Rbs, both S4Δ*aroD* and S4Δ*guaBA* provide protection (≥50% VE) superior to LVS when challenged by aerosol with high doses (~2000 CFU) of S4 [13,14]. Results from vaccine trials with S4Δ*clpB* in a non-rodent system are pending [10]. To date, no side-by-side comparison of these three S4-based candidates in an identical system has been reported.

Licensure of tularemia vaccines will require pivotal efficacy studies in animals because Hu efficacy trials are untenable. In such cases, the Food & Drug Administration (FDA) allows for studies to be performed in animals under the “Animal Rule” which requires that “*the effect is demonstrated in more than one animal species expected to react with a response predictive for humans.*” Inbred Mo are unsuitable models given their well-recognized susceptibility to attenuated *Ft* strains. Rats (Rt) [15,16,17,18,19] and “higher” outbred animals, such as Rb [20,21,22,23] and non-human primates (NHP) [15,24,25,26,27,28], have a more Hu-like susceptibility to *Ft.* However, as we show here, Mo and Rt α-*Ft* antibody (Ab) responses do not fulfill the animal rule because of their striking dissimilarity to Hu α-*Ft* Ab responses. The Animal Rule also requires characterization of the immune response in the model associated with protection. Developing minimally invasive, clinically translatable correlates of protection (COPs) (here defined as pre-challenge metrics that predict, with statistical significance, S4 challenge outcome (survival-S or non-survival-NS)) in a relevant, outbred model will allow extrapolation to predict Hu protection.

One of our goals is to identify humoral COPs applicable to high-dose, aerosol challenge with S4. Following optimization and standardization, such COPs could be used i) to guide future vaccine development choices, and/or ii) to determine which Hu-LVS vaccine recipients are truly protected vs. those that remain at risk. Here, our findings first lead us to conclude that Rbs, but not rodents, are a model appropriate to our goal. A survey of Rb Abs generated by vaccination with the three leading candidates suggests that the percentage of vaccine-induced Ab specific for O-Ag dependent moieties (%O-Ag dep Ab) is related to vaccine efficacy. Analysis of pre-challenge Ab from vaccinated Rbs (that subsequently survived or succumb to S4 challenge) revealed that different vaccines can establish distinct COPs. For S4Δ*guaBA*-vacccinated Rb, ≥70%O-Ag dep Ab is a statistically significant COP and a surrogate marker of survival. This marker was found in 80% of LVS-vaccinated Hu, as expected for a vaccine with 80% efficacy in Hu.

## 2. Results

### 2.1. O-Ag Dependent Ab Reactivity in Vaccinated Humans, Rabbits, Rats and Mice

**Figure 1:**
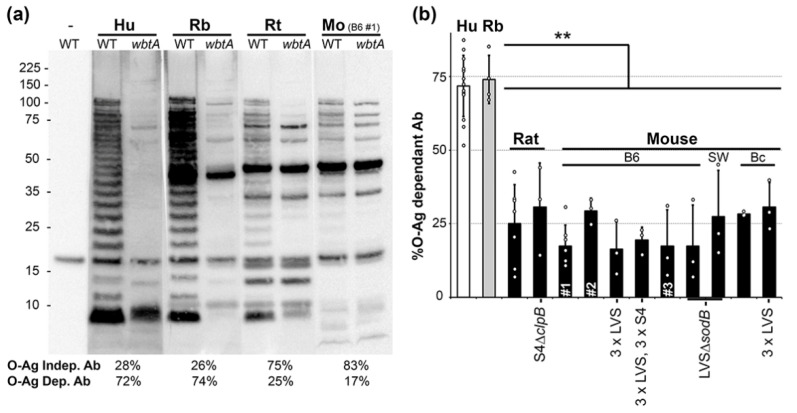
**O-Ag dependent Ab reactivity in vaccinated Hu, Rb, Rt, and Mo.** (**a**) Sera pooled from LVS-primed Hu (n = 10), Rb (n = 5), Rt (n = 5), and C57/B6 Mo (n = 5) were used to probe WT and *wbtA* LVS. “-” indicates no Ab control; the visible band is the biotinylated *Ft* protein AccB detected, even in the absence of *Ft*-specific Ab, by the streptavidin-HRP conjugate. Total protein staining of WT and *wbtA* lysates transferred to nitrocellulose membranes is shown in Figure S1. (**b**) O-Ag dep Ab (means and standard deviations) derived from ≥3 technical repeats (circles) for each of the 13-independent pools of sera/plasma. Each of the 11 rodent pools (black bars) were derived from animals (n = 5–10) primed with LVS, unless otherwise indicated. From the left, the order of the bars are: Hu-LVS (white bar), Rb-LVS (grey bar), Rt-LVS, Rt-S4Δ*clpB*, C57/B6 (B6)-LVS, B6-LVS, B6-3 × LVS (prime-boost-boost), B6-3 × LVS followed by 3 × S4, B6-LVS, B6-LVSΔ*sodB*, Swiss Webster (SW)-LVSΔ*sodB*, Balb/c (Bc)-LVS, Bc-3 × LVS.

As a foundation for our goal of identifying clinically relevant, humoral COPs, we first sought to determine which animal model most closely mimics the Hu Ab response to *Ft* exposure. Previously, we and others had noted that Mo and Hu plasma Ab responses to *Ft* LPS differ substantially [29,30]. To broaden the scope of these reports, we further evaluated the LPS-seroreactivity of Hu, Rb, Rt, and Mo primed with LVS ~30d prior to plasma collection. Plasma pools were used to simultaneously probe lysates of wildtype (WT) and *wbtA* (deficient for O-Ag, the sugar polymer in LPS) *Ft* LVS by Western blot. This approach reveals Abs that bind directly to O-Ag moieties (O-Ag specific Ab), and potentially, Ab that bind epitopes of outer membrane (OM) proteins whose conformation is O-Ag dependent. For each plasma, densitometric analysis of the WT and *wbtA* lanes were used to calculate the percentage of plasma Ab dependent on O-Ag. As shown in Figure 1a, Hu and Rb plasmas both revealed profound differences between the WT and *wbtA* lysates. By densitometry, we calculated that ~25% of the total Ab bound by WT lysates was also bound by the *wbtA* lysates, such that ~75% of the total plasma Ab was O-Ag dependent (Figure 1b). In contrast, with rodent plasmas (Rt, Mo), the WT vs. *wbtA* differences were minimal and the %O-Ag dep Ab were ~25% (Figure 1a,b).

Similar results were obtained with nine additional rodent plasma pools, including multiple (i) genotypes (inbred: Balb/c Mo, C57BL/6 Mo, Fischer 344 Rt; outbred Swiss-Webster Mo), (ii) vaccine strains (LVS, S4Δ*clpB*, LVSΔ*sodB*), and (iii) inoculation regimens (prime, prime-boost-boost (3×), or 3× followed by 3 S4 challenges) (Figure 1b). Collectively, these results indicate that (**a**) the Rb model closely mimics the Hu α-O-Ag antibody response to *Ft* whereas rodent models do not, and (**b**) that “%O-Ag dep Ab” is a metric worthy of further analysis.

### 2.2. O-Ag Dep Ab Reactivity in Rabbits Vaccinated with S4ΔclpB, S4ΔguaBA, or S4ΔaroD

**Figure 2:**
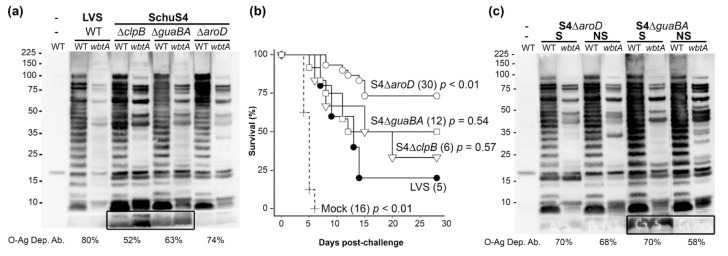
**%O-Ag dep Ab and S4-challenge survival among vaccinated Rbs**. (**a**) Pooled plasma, drawn pre-challenge (d42) from 5 LVS-, 6 S4Δ*clpB*-, 6 S4Δ*guaBA*-, and 12 S4Δ*aroD*-primed (d0) and boosted (d14) Rbs, were used to probe lysates of WT and *wbtA Ft* LVS. Total, and specific, protein detection in WT and *wbtA* lysates is shown in Figure S2 (**b**) Cumulative survival among 69 vaccinated and control Rbs challenged by aerosol with BHI-grown S4 (d44). S4 challenge (d44) doses were ~ 500 CFU for LVS trials and ~2000 CFU for S4-based vaccines. Portions of the survival data have been reported for Mock (n = 8)-, LVS (n = 5)-, S4Δ*guaBA* (n = 6)-, and S4Δ*aroD* (n = 12)- vaccinated Rbs and are provided here in combination with recent results from Mock (n = 8)-, S4Δ*guaBA* (n = 6)-, S4Δ*aroD* (n = 18)-, and S4Δ*clpB* (n = 6)-vaccinated Rbs. Log-rank tests were adjusted for multiple comparisons; *p* values reflect comparisons with LVS. (**c**) Pre-challenge plasma from S4Δ*guaBA*- and S4Δ*aroD*-vaccinated Rbs were pooled by vaccine type from S and NS and used to probe lysates of WT and *wbtA Ft* LVS.

**Figure 3:**
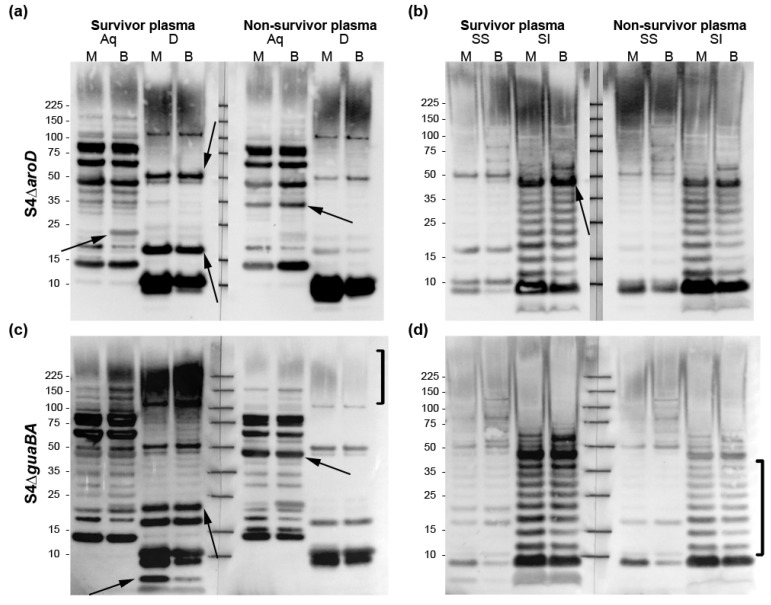
**Blotting of *Ft* fractions with survivor and non-survivor plasmas reveals multiple putative humoral COPs**. *Ft* grown in MHB (M) or BHI (B) were fractionated into Aq, D, SS, and SI phases prior to Western blot analysis with plasma banked from S4Δ*aroD*—(**a**,**b**) or S4Δ*guaBA*—(**c**,**d**) vaccinated Rb. Following S4 challenge completion, S and NS pools were generated from the banked plasmas. Arrows: putative proteins differentially recognized by S and NS Ab. Brackets: O-Ag rich moieties (LPS—SI phase; capsular O-Ag—D-phase) differentially recognized by S4Δ*guaBA* S and NS Ab. See Figure S3 for total protein staining of SDS-PAGE resolved Aq, D, SS, and SI phases.

**Figure 4:**
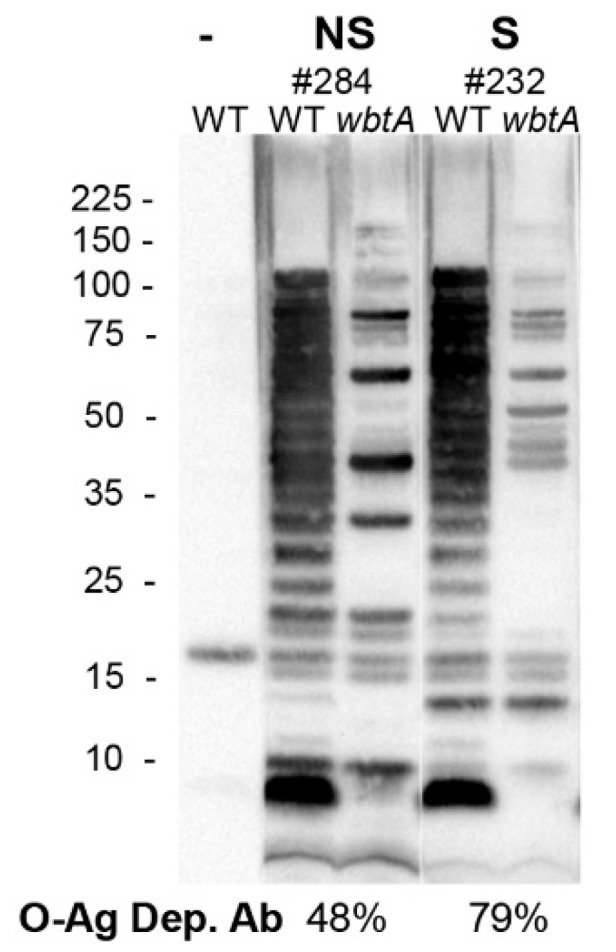
%**O-Ag dep Ab in NS and S** **S4Δ*guaBA*-vaccinated Rbs.** Individual Rb sera were used to probe WT and *wbtA* LVS. Mean %O-Ag dep Ab were derived from >3 repeats. Detection of IglB, IglC, and Tul4 is shown in Figure S4.

Having failed to identify any rodent system that recapitulated the Hu Ab response to *Ft* vaccination (dominated by O-Ag dep Abs), we concluded that Rb were a more appropriate model for identifying Hu-relevant serological COPs. Next, we used this model to determine the %O-Ag dep Ab generated by live-attenuated vaccine candidates with differing vaccine efficacies. For these efforts, Rbs were primed and boosted prior to plasma collection and subsequent aerosol challenge with high doses of BHI-grown S4. Previously, we used this schedule in Rb to demonstrate the vaccine efficacy of LVS (20%), S4Δ*guaBA* (50%), and S4Δ*aroD* (66%) [13,14]. These existing plasmas, along with recent plasmas from S4Δ*clpB*-, as well as additional S4Δ*guaBA*-, and S4Δ*aroD*-vaccinated Rbs were used to probe WT and *wbtA* LVS by Western blot. Initially we present data with plasmas pooled by vaccine type (Figure 2a), subsequently we used plasmas pooled by vaccine type and survival outcome (Figure 2c and Figure 3), and finally plasmas from individual surviving and non-surviving Rbs (Figure 4, Table 1).

For Rb vaccinated with LVS, the %O-Ag dep Ab was not markedly different between animals that received a single inoculation and those that were boosted, 72% and 80% respectively (Figure 1 and Figure 2). Among the S4-based vaccines, the %O-Ag dep Ab ranged from 52% (S4Δ*clpB*), to 63% (S4Δ*guaBA*), to 74% (S4Δ*aroD*). Interestingly, both the S4Δ*clpB*- and S4Δ*guaBA*-induced Ab reacted with a very low MW, O-Ag independent species (Figure 2a, box) that was unreactive with LVS- and S4Δ*aroD*-induced Ab. When the %O-Ag dep Ab values were considered in light of our long-term cumulative survival data (Figure 2b), we noted a trend in which the vaccine efficacy of the S4-based candidates trended with the %O-Ag dep Ab induced by the vaccination. Specifically, vaccination with S4Δ*clpB* induced 52%O-Ag dep Ab and provided 33% protection, vaccination with S4Δ*guaBA* induced 63%O-Ag dep Ab and provided 50% protection, S4Δ*aroD* vaccination induced 74%O-Ag dep Ab and provided 73% protection. These observations suggest that %O-Ag dep Ab in higher animals might be a general predictor of vaccine function among S4-based candidates. We next sought to determine if %O-Ag dep Ab could predict survival *within* a single vaccination group. The ≥50% vaccine efficacy in S4Δ*guaBA*- and S4Δ*aroD*-immunized Rb provided sufficient survivors (S) and non-survivors (NS) to generate plasma pools comprised exclusively of S or NS Ab for each vaccine group. For the S4Δ*aroD* plasma, the %O-Ag dep Ab did not differ between S and NS (70% vs. 68%, Figure 2c). In contrast, the values in S4Δ*guaBA* S plasma (70%) were higher that of the S4Δ*guaBA* NS plasma (58%).

With these plasmas, we noted several O-Ag independent bands (presumably proteins) of reactivity within each vaccine type that were restricted to either the S or NS pools (Figure 2c). To delve more deeply into the *Ft* Ags differentially recognized by NS or S plasma, we fractionated WT LVS (grown in either MHB or BHI to broaden the Ag repertoire) into Tx114 aqueous (Aq) and detergent (D) phases while the Tx-insoluble material was fractioned further into sarkosyl-soluble (SS) and sarkosyl-insoluble (SI) components. This approach partitions the O-Ag rich moieties—high MW capsular O-Ag and LPS—into the D and SI phases, respectively, thereby allowing detection of additional Ags [31,32]. We observed that most dominant protein Ags were commonly recognized by S and NS plasma from S4Δ*aroD* and S4Δ*guaBA* (Figure 3); however, close examination revealed several Ags that were differentially recognized by S and NS Ab (Figure 3, arrows). Several of these Ags were recognized commonly by S4Δ*aroD* and S4Δ*guaBA,* while others appeared unique to a single vaccine type. Characterization of these protein species will be described elsewhere.

Among the O-Ag rich moieties—capsular O-Ag (in the D phases) and LPS (in the SI phases)—we did not observe differences between S and NS Ab in the S4Δ*aroD* plasmas (Figure 3a,b). However, the S4Δ*guaBA* survivor and non-survivor Abs revealed striking differences in recognition of O-Ag capsular material and LPS (Figure 3c,d, brackets). A low MW species (potentially un-glycosylated lipid A) recognized exclusively by survivor S4Δ*guaBA* plasma was observed primarily in the D phase of MHB-grown *Ft* (Figure 3c bottom arrows). These findings, based on WT (non-mutant) *Ft*, also indicate that elevated α-O-Ag Ab levels are a characteristic of survival in S4Δ*guaBA*-vaccinated Rbs.

### 2.3. O-Ag Dep Ab Is a Correlate of Protection in S4ΔguaBA-Vaccinated Rabbits

Next, we determined the %O-Ag dep Ab for plasmas from individual S4Δ*guaBA*-vaccinated Rb; an example is shown in Figure 4 (also see Figure S4 in the Supplementary Materials). When multiple NS (n = 6) and S (n = 6) S4Δ*guaBA* plasma were assayed, the average %O-Ag dep Ab values were 60% for NS and 76% for S. These differences were significant by the Wilcoxon Rank Sum test (WRS, *p* = 0.014). The anti-*Ft* ELISA-determined titers for the S (1575 ± 950) and the NS (1994 ± 1213) were not significantly different. Receiver operating characteristic (ROC) analysis is used to evaluate the utility of a measurement for predicting an outcome. ROC analysis of %O-Ag dep Ab as a classification model for survival yielded an AUC = 0.94, 95% CI (0.82, 1.00). A cut-off of 70% yields 83% sensitivity and 83% specificity for predicting challenge survival. Thus, an elevated (>70) %O-Ag dep Ab is a statistically significant COP for S4Δ*guaBA*-vaccinated Rbs.

### 2.4. O-Ag Dep Ab in LVS-Vaccinated Humans

**Figure 5:**
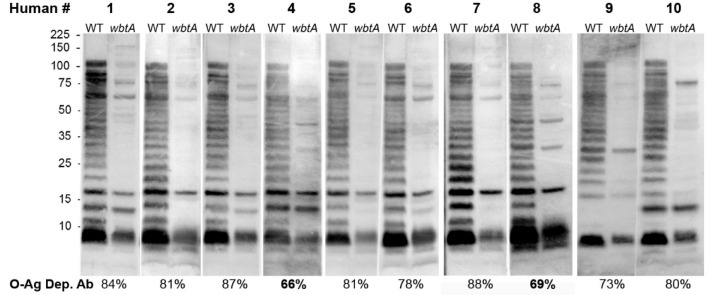
%**O-Ag dep Ab in LVS-vaccinated Hu**. Individual sera from LVS-vaccinated Hu were used to probe WT and *wbtA* LVS. Mean %O-Ag dep Ab were derived from ≥3 repeats with each sera. Total protein detection in WT and *wbtA* lysates is shown in Figure S5.

As our Rb data suggests that >70 %O-Ag dep Ab is a COP, we sought to determine if this metric had relevance to Hu. Currently there are no plasma from S4Δ*guaBA-*vaccinated Hu. There are, however, (**i**) banks of plasma from LVS-vaccinated Hu, and (**ii**) data indicating that LVS vaccination of Hu is ~80% effective in preventing disease induced by low doses (~50 CFU) of S4 [5,6]. Based on the rationale that *bona fide* COPs should therefore be present in ~80% of LVS-vaccinated Hu plasma, we assessed Hu plasma drawn from 10 LVS-vaccine recipients ~d30 post-vaccination. The %O-Ag dep Ab among the Hu ranged from 66–88% (Figure 5) with a mean of 79%. Only two of these plasmas (#4 and #8) had O-Ag dep Ab values below 70%. The remaining 80% had higher values, reminiscent of S4Δ*guaBA*-vaccinated Rbs that went on to survive S4 challenge. It is worth noting that %O-Ag dep Ab is not predicted by simple α-*Ft* ELISA titer analysis. The Hu plasma having the highest titers (1280 and 2560) did not stand out in the %O-Ag dep Ab assays. Similarly, plasma (#4 and 8) had the same titers (320–640) as many other plasma (#2, 3, 7, and 9) that did not stand out. Thus, this COP, developed using S/NS Rbs and present in vaccinated Hu, appears to be a novel metric providing distinct information that augments and/or surpasses anti-*Ft* Ab titer data obtained by ELISA.

## 3. Discussion

Defining COPs for rare infectious diseases is not trivial, as establishing protection involves willingly infecting humans; in the case of highly lethal pathogens this is not possible in modern times. In the Cold War era, protection afforded by immunization with LVS was determined by challenging vaccinated Hu with aerosols of S4 and monitoring for overt clinical disease (a lack of protection which cued antibiotic intervention) [2,5,6]. Collectively, these Hu studies quantified LVS-mediated protection against low dose S4 challenge as ~80%. However, despite the efforts of multiple groups, no COPs were identified, such that 20% of Hu LVS vaccinees remain unprotected and unidentified. Moreover, the lack of Hu COPs has continued to hinder development of novel tularemia vaccine candidates.

To circumvent the limitations of Hu-based research, animals have been used as surrogates in efforts to identify COPs, both cellular [33,34,35,36,37,38,39,40,41,42] and humoral [33,36,43,44]. Most frequently, these efforts have involved immunizing rodents with vaccine candidates that provide varying degrees of protection and then analyzing induced responses (cells, cytokines, antibodies, etc.) of the differentially immunized groups. We similarly sought to ID clinically-useful, serological COPs but planned to bank individual vaccine-induced Ab samples for retrospective analysis after each animal’s S4 challenge outcome—survival or non-survival—was known. As a foundation for these studies, we compared the serum Ab responses of LVS-vaccinated mice, rats, and Rbs, to that of humans to inform our choice of animal model. By simultaneously assessing the reactivity of Ab samples with O-Ag sufficient and deficient *Ft*, we were able to determine the percentage of vaccine-induced Ab response that was dependent on O-Ag. This internally controlled approach allows for the comparison between plasma/sera of different titers and across species. While differences between Mo and Hu α-LPS responses have been reported [29,30], the results here from side-by-side quantification of rodent, Rb, and Hu α-O-Ag Ab responses to LVS immunization were none-the-less, quite striking. Hu and Rb responses were dominated by O-Ag-dep Ab (~75% of the total *Ft*-specific Ab), whereas in rodents the same measure was ~25%. Multiple attempts to increase this value in rodents included (**i**) varying the strain of inbred rodent, (**ii**) the use of outbred rodents, (**iii**) alteration of the vaccine regimen/route, and (**iv**) the use of distinct vaccine candidates. Collectively, our data suggest a fundamental discord between rodents and higher animals regarding the O-Ag dep Ab responses to *Ft* infection. In this regard, our findings are conceptually very similar to those of Rahhal et al., in which Hu B cells were found to be significantly more responsive than Mo B cells to purified *Ft* LPS [45]. Our data do not mean that rodents fail to produce α-LPS Ab following *Ft* infection, just that they do so to a much lesser extent—with a greater focus on protein Ags—than higher animals such as Rb and Hu.

The mechanism(s) underlying this difference is unknown to us, as is the extent to which this difference may also apply to other Gram-negative infections. All the rodents in our study were purchased and maintained under SPF (specific pathogen free) conditions and such abnormally hygienic conditions impact development of the immune system [46]. The Rbs in our studies were also purchased and maintained under SPF conditions, although the qualifications for SPF rodents and SPF Rbs are not identical. Thus, while differences in the “cleanliness” of SPF-rodents, SPF-Rbs, and adult Hu likely contribute to our findings, other factors such as germ line, genotypic differences [47] cannot be excluded.

Operationally, regardless of the mechanism(s), these findings revealed that Rbs were the animal of choice in which to search for Hu-relevant, serological COPs. When rabbits were immunized with different strains, we found that vaccine-induced %O-Ag dep Ab (52–74%) trended with the vaccine efficacy (33–73% respectively) for S4-based strains. However, vaccination with LVS was poorly protective (20%) despite yielding a high %O-Ag dep Ab (80%). To account for the above results, we envisioned two possible explanations: (**i**) %O-Ag dep Ab can act as a COP only when combined with another type/facet of immunity (preferentially provided by S4-based vaccines), or (**ii**) %O-Ag dep Ab is a correlate for some, but not all, S4-based *Ft* vaccines. To discern these possibilities, we characterized responses of survivors and non-survivors emanating from immunization with two vaccine strains (S4Δ*aroD* and S4Δ*guaBA*) that provided ≥50% protection. Collectively, the antigenic differences between S4Δ*aroD*-vaccinated S and NS appeared to involve a small set of protein Ags (Figure 3), but no discriminatory involvement of O-Ag moieties (both S4Δ*aroD* S and NS have ~70% O-Ag dep Ab). In contrast, S4Δ*guaBA-*vaccinated S and NS were strongly differentiated by %O-Ag dep Ab, with lesser discrimination by protein Ags. Thus, it appears that the protective immunity provided by S4Δ*aroD* is mechanistically different than the protective immunity provided by S4Δ*guaBA*. The low survival rate among LVS- and S4Δ*clpB*-vaccinated Rb challenged with high doses of S4 precluded characterization of the infrequent protective immunity/COPs for these strains. The notion that different vaccines targeting a single infectious agent could have distinct mechanisms of protection and distinct COPs is well-established [48].

While the goal of this work was to identify correlates of protection, and correlation does not mean causation, we can envision a mechanism by which high %O-Ag dep Ab could reflect effector functions that contribute to the survival of S4Δ*guaBA*-vaccinated Rbs challenged with S4. While unopsonized *Ft* replicate exponentially in host MΦs, *Ft* opsonized with saturating concentrations of complement or α-LPS Ab gain cellular entry through phagocytic receptors (complement- and/or Fc- receptors) and show limited intracellular replication [49]. Thus, in vaccinated higher animals (which are capable of a mounting a robust α-OAg Ab response), a sufficiently O-Ag-focused Ab response would lead to opsonization of an S4 challenge bolus with Ab (and classically-activated C’). The resulting restricted intracellular replication could allow time for additional vaccine-induced effectors (cytokines, cell-mediated immunity, etc.) to engage. In this scenario; %O-Ag dep Ab could be considered either a mechanistic COP or potentially, a co-correlate [48]. Opsonophogocytosis assays with Rb S and NS sera are underway to determine the impact of these sera on intracellular growth of *Ft*. In the context of S4Δ*guaBA*-vaccinated animals, our Rb sera fits nicely in the above rationale, as S had significantly higher %O-Ag dep Ab than NS, such that >70%O-Ag dep Ab is a statistically significant indicator of survival. Moreover, this notion also fits with our findings that vaccination of mice (which mount limited α-LPS Ab responses) with S4Δ*guaBA* is not protective [12]. While the above scenarios describe a positive correlate/activity that is preferentially present in the survivor samples, it is also theoretically possible that a negative correlate/detrimental activity is overly abundant in the NS samples. Both the S4Δ*guaBA* and S4Δ*aroD* NS sera samples displayed Ab reactivities with proteins that were non-reactive with the corresponding S sera (Figure 2, Figure 3 and Figure 4). Identification of these *Ft* proteins is in progress.

Formally proving that a candidate COP (such as %OAg-dep Ab, or Ab reactivity with specific *Ft* protein Ags) is Hu-relevant is daunting, as Hu-vaccination and challenge studies ended in the 1960s. However, sampling individual vaccine-induced Hu responses (when the level of vaccine-mediated protection is known) can exclude candidate responses as COPs if their frequency is markedly different from the known frequency of protection. In other words, a true Hu-relevant COP should be present in ~80% of the Hu LVS vaccinee population. Responses that are present in 20% (or 100%) of the Hu LVS vaccinee population are likely not true COPs. In this light, we surveyed a small set of Hu LVS vaccinee sera for the Rb-derived COP “≥70% O-Ag dep Ab”. Our observation, that 80% of Hu had this COP, while 20% did not, indicates that this marker is a putative Hu-relevant COP and is worthy of further analysis. We have arraigned for a larger sample size of Hu LVS sera for such analysis; however, definitive proof of these COPs as primate-relevant will require non-human primate (NHP) vaccination and challenge studies along the line of our Rb studies. Given the high protection rates of S4Δ*aroD* and S4Δ*guaBA* in Rbs (50 and 73% respectively), we envision that NHP studies with these strains would both advance tularemia vaccines and further test the notion that Rb-derived COPs have clinical utility.

## 4. Materials and Methods

### 4.1. Bacteria

The LVS strain of *Ft* was acquired from BEI (NR-646) and was grown in vitro in MHB or BHI as previously described [31,32]. The LVS *wbtA* mutant similarly used here has been described [50]. The *clpB* mutant of S4 [10] was provided by the National Research Council-Canada. The RML isolate of LVS was provided by Catharine Bosio. Plasmid-modification of *Ft* was by electroporation [51]. WT *Ft* S4 was used within the CDC-certified A/BSL-3 facilities of Albany Medical College or the University of Pittsburgh.

Late-log phase *Ft* grown in MHB or BHI were harvested by centrifugation (8000× *g*, 15 min, 20 °C). Bacterial pellets were resuspended in the appropriate fresh growth media, transferred to pre-weighed Eppendorf tubes, pelleted as above after which the transfer supernatant was aspirated. Pellet wet weights were determined and used to estimate cell numbers based upon the estimate of 1 mg of wet weight = 5 × 10^8^ bacteria. Cells were resuspended to 5 × 10^7^/μL in 20 mM Tris, pH 8.0 containing 100 mM NaCl, 20 μL/mL protease inhibitor cocktail #P8849 (Sigma, St Louis, MO, USA). These whole cell (WC) preparations were either used for SDS PAGE and western blot analysis (below) or first fractionated into Aq, D, SS, and SI phases as previously described [29].

### 4.2. SDS-PAGE and Western Blot Analysis

Samples derived from 10 μg of *Ft* protein (~1 × 10^8^ cells) were mixed with Laemmli sample buffer and boiled for 10 min prior to resolution through 4–12% gradient SDS-PAGE pre-cast gels (Invitrogen, Waltham, MA, USA). The running buffer was NuPAGE MES SDS buffer from Invitrogen; gels were variously run at 90–160 V. Resolved gels were stained with either Coomassie blue (BioRad, Hercules, CA, USA) or transferred to nitrocellulose membranes. Coomassie-stained gels were scanned into Adobe Photoshop using an HP 2820 scanner. Membranes were blocked for 30 min with PBS, 0.05% Tween 20, 5% non-fat dry milk. Primary Abs were applied for overnight incubation at dilutions ranging from 1:1000 to 1:5000. The secondary Ab was a biotinylated goat α-species-specific Ab (1:1000 to 1:5000) followed by a tertiary conjugate of streptavidin-linked HRP (1:1000 to 1:5000). Development of the chemiluminescent substrate (SuperSignal West Pico and Femto, (Pierce, Rockford, IL, USA) was visualized using a BioRad ChemiDoc Touch imaging system in movie mode. Densitometric analysis of developed blots was performed on the same system. Antibodies specific for the *Ft* proteins IglC, IglC, FopA, and Tul4 have been described previously [32].

For blots in Figure 1, Figure 2, Figure 4 and Figure 5: multiple, alternating lanes were loaded with whole cell lysates of WT and *wbtA* LVS, resolved by SDS-PAGE and transferred to a nitrocellulose membrane. Sections of the membrane, each containing one lane of LVS and one lane of *wbtA*, were probed with one of the indicated plasma or sera. Except for differences in the primary antibody and species–specific secondary Ab, all membrane sections within each figure were developed under identical conditions.

### 4.3. Densitometric Determination of %O-Ag Dep Ab

Densitometric analysis of developed blots was performed on BioRad Image Lab 6.0 Software (BioRad, Hercules, CA, USA) in which auto exposed chemiluminescent images were analyzed via the volume tool. A large rectangle was drawn to encompass 80% of a lane and then duplicated across all lanes and top aligned. Each blot contained a “no-sera control (nsc)” lane in which WT *Ft* lysate was probed with only SA-HRP, or 2° Ab (biotinylated goat IgG anti-species) and SA-HRP. After outlining each lane, the volume table was exported into excel for analysis. The volume generated from the “no-sera control” lane was subtracted from the volumes of the other lanes. For each sera, a volume was calculated for each WT lysate and *wbtA* lysate. %O-Ag dep Ab = 100 − [Vol(*wbtA* − nsc)/Vol(WT-nsc) × 100]. Each sera sample was analyzed by at least three independent Western blots.

### 4.4. Sera/Plasma

With three exceptions detailed below (Rt α-LVS, Rt α-S4Δ*clpB*, and Rb α-S4Δ*clpB*), the generation/provision of all sera has been previously described. Mice: 2 groups of LVS primed C57BL/6 [51]; BalbC 1×, 3×; B6 3× and 3 × LVS/3 × S4 [29]; LVSΔ*sodB* vaccinated C57BL/6 and Swiss Webster [34]. Rabbits: LVS [13], S4Δ*aroD* and S4Δg*uaBA* [14]. Humans: [29]. Rat LVS and S4Δ*clpB* antisera*:* All rat studies were approved by MRIGlobal’s Institutional Animal Care and Use Committee (AUS 16–33) and by the U.S. Army Medical Research and Material Command’s Animal Care and Use Review Office. Fischer (CDF) 344/SuCrl (strain code 002) rats (equal mix male and female) were obtained from Charles River Laboratories (Wilmington, MA). LVS used for rat vaccinations was manufactured by the Dynport Vaccine Company and was diluted to a concentration of 2 × 10^6^ colony forming units (cfu)/mL in phosphate buffered saline (PBS). S4Δ*clpB* was prepared at National Research Council-Canada shipped to MRI Global. S4Δ*clpB* was diluted to a concentration of 2 × 10^6^ cfu/mL in PBS. Rats were shaved on the right flank and 50 µL of vaccine was injected to administer a target dose of 1 × 10^5^ cfu/rat. Blood was collected from the jugular vein or cranial vena cava 25 days after vaccination. Blood was processed to serum and transferred to Albany Medical College for analysis. Rb S4Δ*clpB* antisera: Young female SPF New Zealand White Rbs (Robinson Services, Inc., Malden, MA, USA) were housed in the University of Pittsburgh Regional Biocontainment Laboratory (RBL) at ABSL-3. All studies were approved by the University of Pittsburgh’s Institutional Animal Care and Use Committee (Protocol # 19014420). Rb were primed (d0) and boosted (d14) with BHI-grown S4Δ*clpB* delivered by aerosol using a Collison 3-jet nebulizer and aerosol sampling with an all-glass impinger, as previously described for vaccination with BHI-grown S4Δ*aroD* and S4Δg*uaBA* [14]. Aerosol exposures were performed inside a class III biological safety cabinet in a dedicated Aerobiology suite in the University of Pittsburgh Regional Biocontainment Laboratory and controlled by a Aero3G aerosol exposure system (Biaera Technologies, Hagerstown, MD). Delivered doses of S4Δ*clpB* were confirmed by plate-counting as 1.4 × 10^8^ CFU (prime) and 1.0 × 10^8^ CFU (boost). Rb α-S4Δ*clpB* sera used in this manuscript were drawn on day 42 (28 post-boost). Boosted Rb were challenged by aerosol on d44 with 2.1 × 10^3^ CFU of BHI-grown S4 and monitored twice daily for 28 d (until d72).

### 4.5. Statistics

Statistically significant differences were calculated by either GraphPad Prism 5 or Microsoft Excel. Differences in *O-Ag* dep Ab were determined by two-way ANOVA or *t*-test. *p*-values < 0.05 were considered significant. Percent O-Ag dependence are presented as the means and standard deviations. Rb survival: Time-to-event data was analyzed using standard survival techniques with days until sacrifice as the primary endpoint. Subjects were censored at the last day of follow up (Day 28). Technical replicates from assays were averaged within individual and two-sided non-parametric tests were used. A significance level of 0.05 was used for all analyses and *p*-values were adjusted for multiple comparisons (Benjamini-Hochberg method) for post-hoc tests across multiple groups. Receiver operating characteristic (ROC) curves were used to determine cut-off values for predicting survival. All analyses were performed using Rstudio (copyright 2009–2021) Version 1.4.1106 (RStudio Software company, Boston, MA, USA).

## Figures and Tables

**Table 1:** %O-Ag dep Ab is a COP for S4Δ*guaBA*-vaccinated Rbs.

	NS	S
**Range**	43–70%	68–81%
**Mean**	60%	76%
**WRS**	*p* = 0.009	
**ROC**	AUC = 0.94, 95% CI (0.82, 1.00) 70% cut-off = 83% Sensitivity, Specificity.	

## Data Availability

All pertinent results are contained within the article or supplement.

## Supplementary Materials

The following are available online https://www.mdpi.com/article/10.3390/pathogens10121646/s1. Figure S1. Amido black total protein staining of WT and wbtA lysates transferred to nitrocellulose membranes; Figure S2. Detection of total and specific proteins in WT and wbtA LVS; Figure S3. Ft grown in MHB (M) or BHI (B) were fractionated into Aq, D, SS, and SI phases prior to resolution by SDS-PAGE and total protein staining with SYPRO Ruby Protein Gel Stain; Figure S4. Detection of IglB, IglC, and Tul4 in WT and wbtA LVS using a cocktail of 3 mAbs specific for these Ft proteins; Figure S5. Amido black total protein staining of WT and wbtA lysates transferred to a nitrocellulose membrane prior to being cut into discrete sections for subsequent incubation with sera/plasma.
