# Supplementary material for: The O-Ag Antibody Response to Francisella Is Distinct in Rodents and Higher Animals and Can Serve as a Correlate of Protection
Source: Pathogens. 2021 Dec 20;10(12):1646. doi: 10.3390/pathogens10121646 (PMC8704338; doi:10.3390/pathogens10121646)
Supplement: Supplementary file 1 [file pathogens-10-01646-s001.zip › pathogens-1479556-supplementary.pdf]

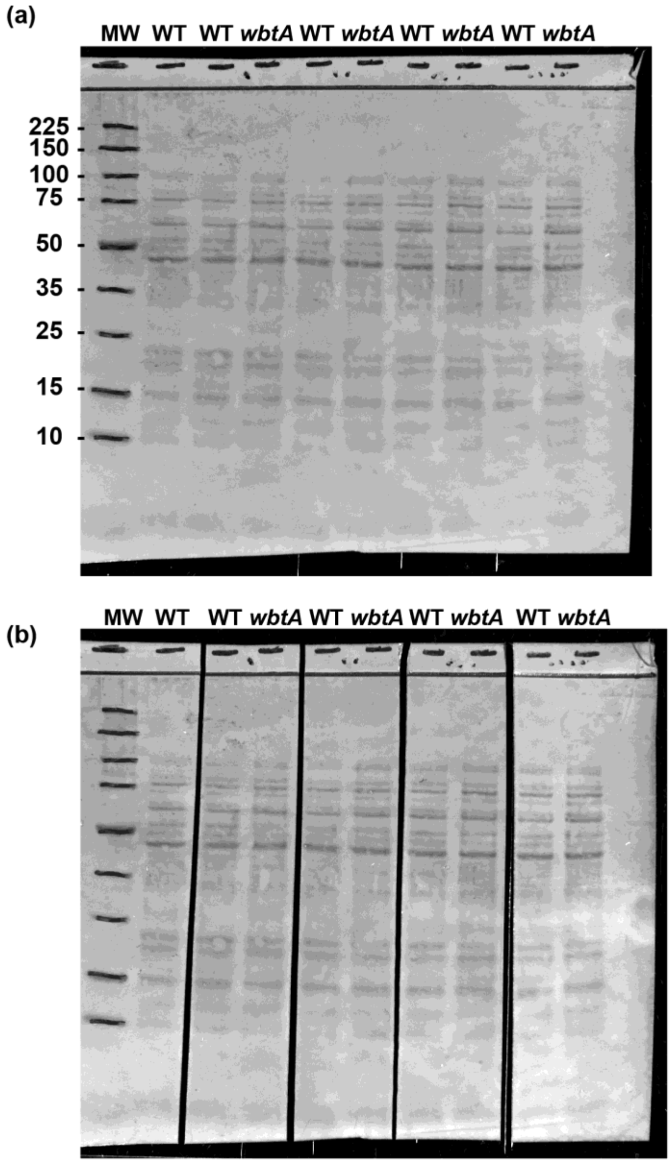


**Figure S1.** Amido black total protein staining of WT and *wbtA* lysates transferred to nitrocellulose membranes. Panel (**a)** shows the intact, stained membrane. Panel (**b)** shows the same membrane cut into discrete sections. The section containing the MW and a single lane of WT was not exposed to any Abs. The remaining sections, each containing a WT and *wbtA* lane, were subsequently incubated with a different sera/plasma as the primary Ab. The secondary Abs were biotinylated goat anti-species IgG, and lastly all sections were exposed to Streptavidin (SA)-linked-HRP and aligned for chemiluminescent development shown in **Fig 1**. SA-HRP binds to AccB, an endogenously biotinylated *Ft* protein of ~18 kDa as well as any bound 2' biotinylated Ab.

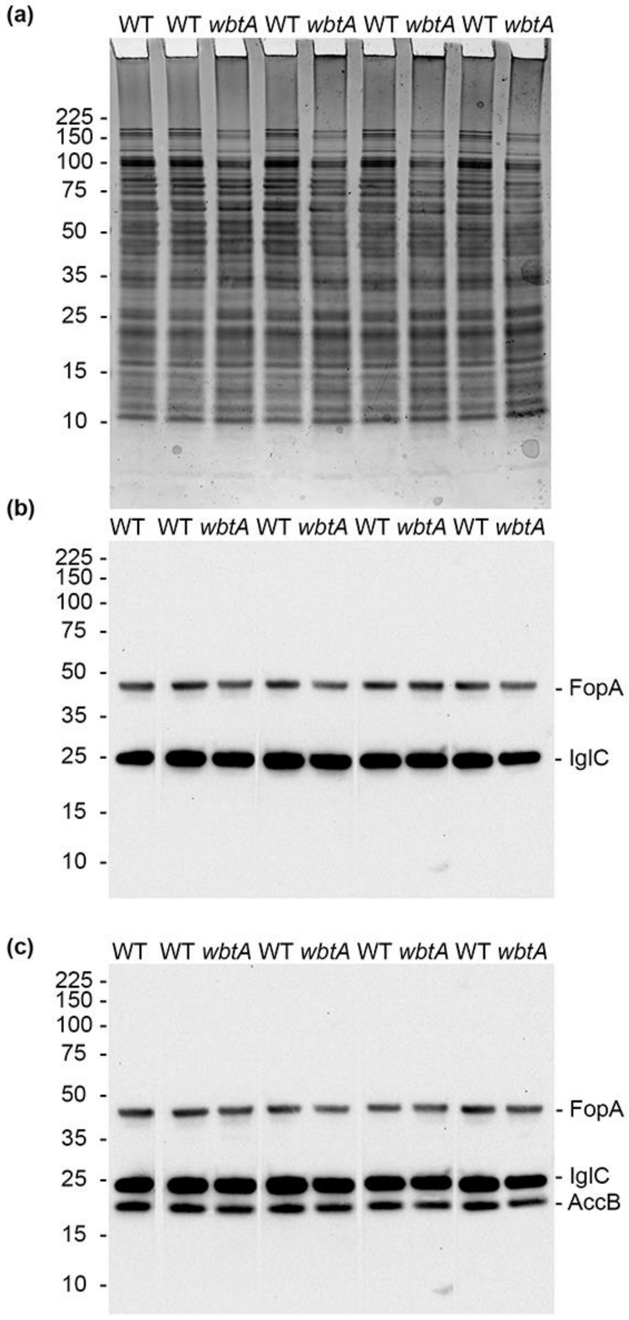


**Figure S2.** Detection of total and specific proteins in WT and *wbtA* LVS. Lysates were resolved by SDS-PAGE prior to **(a)** staining with Coomassie blue or transferred to a nitrocellulose membrane and cut to sections as in Fig S1b. The sections were incubated in a cocktail of murine primary Ab against the *Ft* proteins FopA and IglC. Following incubation with HRP-linked goat anti-mouse, the membranes were developed for chemiluminescent detection shown in panel **(b).** Following the imaging, the sections were incubated in Streptavidin-linked HRP which binds to AccB, an endogenously biotinylated *Ft* protein. The membranes were then re-developed for chemiluminescent detection shown in panel **(c)**.

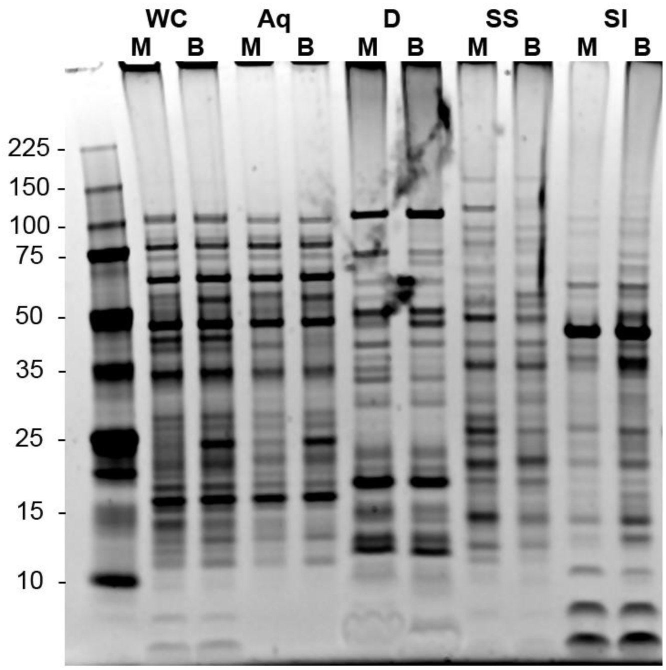


**Figure S3.** *Ft* grown in MHB (M) or BHI (B) were fractionated into Aq, D, SS, and SI phases prior to resolution by SDS-PAGE and total protein staining with SYPRO Ruby Protein Gel Stain.

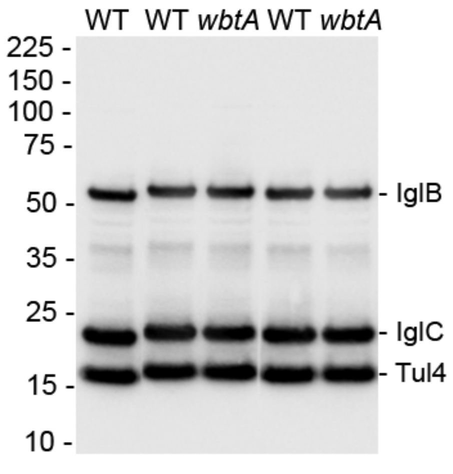


**Figure S4.** Detection of IglB, IglC, and Tul4 in WT and *wbtA* LVS using a cocktail of 3 mAbs specific for these *Ft* proteins.

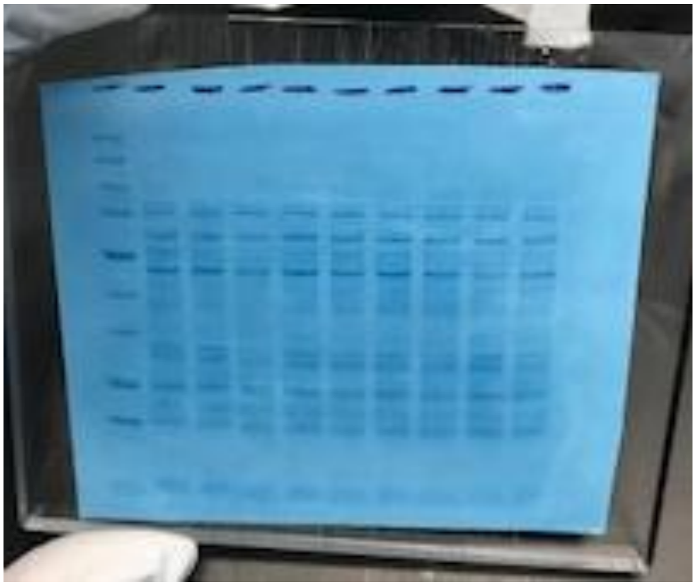

**Figure S5.** Amido black total protein staining of WT and *wbtA* lysates transferred to a nitrocellulose membrane prior to being cut into discrete sections for subsequent incubation with sera/plasma.
